# Barriers and facilitators related to implementation of regulated midwifery in Manitoba: a case study

**DOI:** 10.1186/s12913-016-1334-5

**Published:** 2016-03-15

**Authors:** Kellie Thiessen, Maureen Heaman, Javier Mignone, Patricia Martens, Kristine Robinson

**Affiliations:** College of Nursing, Faculty of Health Sciences, University of Manitoba, 89 Curry Place, Winnipeg, MB R3T 2N2 Canada; Department of Community Health Sciences, Faculty of Health Sciences, University of Manitoba, Winnipeg, MB R3T 2N2 Canada; Department of Community Health Sciences, College of Medicine, Faculty of Health Sciences, University of Manitoba, 408-727 McDermot Avenue, Winnipeg, MB R3E 3P5 Canada; Winnipeg Regional Health Authority, Clinical Nurse Specialist St. Boniface General Hospital, 409 Tache Avenue, Winnipeg, MB R2H 2A6 Canada

**Keywords:** Midwifery, Implementation, Utilization, Barriers, Facilitators, Case study, Manitoba, Canada

## Abstract

**Background:**

In 2000, midwifery was regulated in the Canadian Province of Manitoba. Since the establishment of the midwifery program, little formal research has analyzed the utilization of regulated midwifery services. In Manitoba, the demand for midwifery services has exceeded the number of midwives in practice. The specific objective of this study was to explore factors influencing the implementation and utilization of regulated midwifery services in Manitoba.

**Methods:**

The case study design incorporated qualitative exploratory descriptive methods, using data derived from two sources: interviews and public documents. Twenty-four key informants were purposefully selected to participate in semi-structured in-depth interviews. All documents analyzed were in the public domain. Content analysis was employed to analyze the documents and transcripts of the interviews.

**Results:**

The results of the study were informed by the Behavioral Model of Health Services Use. Three main topic areas were explored: facilitators, barriers, and future strategies and recommendations. The most common themes arising under facilitators were funding of midwifery services and strategies to integrate the profession. Power and conflict, and lack of a productive education program emerged as the most prominent themes under barriers. Finally, future strategies for sustaining the midwifery profession focused on ensuring avenues for registration and education, improving management strategies and accountability frameworks within the employment model, enhancing the work environment, and evaluating both the practice and employment models. Results of the document analysis supported the themes arising from the interviews.

**Conclusion:**

These findings on factors that influenced the implementation and integration of midwifery in Manitoba may provide useful information to key stakeholders in Manitoba, as well as other provinces as they work toward successful implementation of regulated midwifery practice. Funding for new positions and programs was consistently noted as a successful strategy. While barriers such as structures of power within Regional Health Authorities and inter and intra-professional conflict were identified, the lack of a productive midwifery education program emerged as the most prominent barrier. This new knowledge highlights issues that impact the ongoing growth and capacity of the midwifery profession and suggests directions for ensuring its sustainability.

## Background

Throughout history, midwives have taken on many titles based on their training and geographic location. The most commonly recognized titles in the profession are traditional birth attendant (TBA), skilled birth attendant (SBA), lay midwife, direct-entry midwife (DEM), certified professional midwife (CPM), certified midwife (CM), registered midwife (RM), and certified nurse-midwife (CNM). Traditional birth attendants (TBAs) are more notably recognized in developing countries and typically have no formal education related to the profession [1]. The legal implications and scope of practice of midwives varies among jurisdictions within each country.

In Canada, the model of midwifery is unique in that midwifery is separate from nursing [2]. In provinces where midwifery is regulated, midwives must be registered with their colleges to use the title of registered midwife (RM). Additionally, the baccalaureate education programs for midwifery in Canada are direct entry, which means no credentials are required prior to entry [3]. While there are vast differences in how midwives become educated and practice around the world, Canada and the United States share some similarities in their models of midwifery. Overall, midwives in both countries are autonomous health providers who provide evidence-based care to women before, during, and after their pregnancy [4, 5] However, nurse midwives are more common in the United States. In spite of the differences in practice, regulation, and education, midwifery around the world shares a core philosophy of facilitating informed choice, respecting women’s right to choose, promoting cultural sensitivity, and emphasizing health promotion [6].

Since the 1990s, midwifery began the process of becoming regulated in various jurisdictions across Canada, with Ontario, British Columbia, Alberta, and Quebec being the first four provinces to be regulated [7]. In 2000, Manitoba was the fifth province to regulate midwifery in Canada. Regulation refers to the legal process whereby the proclamation of the Midwifery Act of 2000 enabled midwifery in Manitoba to be a self-regulated, autonomous profession [8]. The model of midwifery care in Manitoba includes informed choice, continuity of care, and choice of birth setting [9]. The midwives provide full scope of care to clients both in hospital and community based settings during the prenatal and intrapartum period, and up to six weeks postpartum. Midwives may attend births in hospitals where they are required to obtain privileges, and may use a registered nurse as a second birth attendant. Likewise, midwives also see women in their homes for labour and birth, where a second attendant is another midwife. Finally, in Manitoba in 2011, a free-standing birth centre was introduced as a third option for women and their families [10]. The birth centre is midwifery-led and is not located in a hospital. In FSBCs, midwives, along with birth centre aides, provide antenatal, labour and birth, and postpartum care.

Recent and past evidence demonstrate favorable maternal/fetal outcomes related to midwifery and support for out-of-hospital births [11, 12]. For example, the Birthplace in England Collaborative Group (2011) found no difference in perinatal mortality and intrapartum neonatal morbidities between birth settings (hospital obstetric unit, home, or freestanding midwifery unit) [13]. Midwifery care is also associated with lower rates of intervention. One study showed women in the midwifery group had lower rates of episiotomy, 3^rd^ or 4^th^ degree tears than women in the medical group. Cesarean section in both multiparous and nulliparous groups was also lower compared to those receiving care from the medical group [14]. Furthermore, midwifery care has been associated with satisfaction with care [15–17].

Prior to regulation in Manitoba, a Human Resource Strategy Group was formed in 1998 by Manitoba Health to develop implementation strategies to integrate midwifery into the regional health care system; a payment model for the midwives; and a plan for midwifery education with an emphasis on Northern and Aboriginal communities [18]. The goals for implementing the midwifery program were to: ensure women had increased access to primary care from a midwife; target priority populations (single, adolescent under 20 years, immigrant/newcomer, Aboriginal, socially isolated, poor, and other at-risk women); and fully integrate midwives into the Regional Health Authorities around the province [19]. Since regulation of midwifery in 2000, however, some key components of the original implementation plan have not been fully established and the projected targets for the number of midwives and midwifery-attended births have not been achieved. It was projected that 140 midwives would need to be integrated into the Regional Health Authorities by the year 2005 in order to meet the goal that 14 % of all births in the province would be attended by a midwife [18]. Instead, the number of practicing midwives has seen only a modest increase, from 26 in 2001/02 to 40 in 2009/10 [20, 21], falling short of the projected goal of 140 midwives by 2005. Between 2001/02 and 2009/10, midwives attended 4.8 % of all births in the province with the highest proportion of midwifery-attended births reported in 2006 at 5.7 %, again falling short of the projected target of 14 % [22] Table 1 provides a brief synopsis of the targeted goals of the original implementation plan of midwifery in Manitoba in comparison to the status of midwifery as of 2011.

**Table 1:** Comparison of Projected Targeted Goals of the Implementation Plan to 2011 Status

Goals from original implementation plan: Integration into regional health care system, increase access to primary care for women, target priority populations: adolescent (< 20), Aboriginal, immigrant, socially isolated, poor, other (Manitoba Health, 1998).	
Birthrate	Status as of 2011
Midwifery-attended births were to be at 14 % of provincial births within 2.5 years of implementation	Midwifery-attended births (2009/10 data) =5 %
Number of Midwives/Vacancies/Consumer Demand	
Projected Plan: Human Resource Strategy for Midwifery Implementation (1998) projected:	2010: 38 practicing, 15 non-practicing
Within 2.5 years of legislation there would be 50 midwives each attending 40 births = 2000 births.	2010: 45 funded
By 2005 need: Approximately 140 practicing midwives in the province.	Consumer demand: Percentage of women that sought midwifery care and were declined care in 2011:
Originally (2000), 26 fully funded positions	NOR-MAN: 40 %
	Regional Health Authority Central: 55 %
	Winnipeg Regional Health Authority: 70 %
	Brandon: 60 %
Education programs	
Proposal for Bachelor of Midwifery Program at University of Manitoba (1999)	Program was not funded
Aboriginal Midwifery Program implemented by UCN (2006)	11 original students, no graduates, program’s conditional approval was rescinded by College of Midwives of Manitoba in 2011.
Pathways Program implemented by UCN (2009)	12 candidates, 10 accepted into program never implemented
University College of the North, Bachelor of Midwifery Program, southern program (2010)	11 students enrolled; 10 students graduated by 2014
Evaluation framework	
Recommended that Manitoba Health implement a Midwifery Evaluation Advisory committee	Formal evaluation was completed in 2013, information has not been released

Data was obtained from *Human resource strategy midwifery implementation: The Manitoba scene, by* Manitoba Health, 1998. & J. Erikson, personal communication, September 1, 2011

Implementation in the context of Canadian midwifery has been referred to in the literature as the following key components: having a legislative framework, a regulatory body, funding for midwifery positions, and an education program [23, 24]. The question is why key components of the implementation plan have failed to reach the intended goals in Manitoba, thus leading to a shortage of midwives across the province which directly impacts women’s access to this maternity care option. For example, in 2011, 70 % of the women who requested midwifery care in the Winnipeg Regional Health Authority were declined such care because of the full caseloads of the midwives (J. Erikson, Registrar, College of Manitoba Midwives, personal communication, September 1, 2011).

Furthermore, in spite of these on-going challenges, only small initiatives have been implemented to critically evaluate how the program goals and objectives have been met. Other than anecdotal information regarding the shortage of midwives in Manitoba, no critical analysis has been done to explore factors that have influenced the implementation and utilization of regulated midwifery services since the legislation was enacted. Therefore, the purpose of our study was to explore factors influencing the implementation and utilization of regulated midwifery services in Manitoba. This article reports on the key findings that emerged from the following research questions:

1. What barriers and facilitators have impacted the implementation and utilization of regulated midwifery services in Manitoba from 2001/02 to 2009/10?
2. How has the workload and organization of the midwifery profession impacted the utilization of regulated midwifery services in Manitoba from 2001/02 to 2009/10?
3. What strategies need to be implemented to improve utilization of midwifery services in Manitoba?

## Methods

A case study design was used to explore factors that have influenced the implementation of regulated midwifery. Case study research investigates a phenomenon in-depth and in the context of a real-life situation [25]. In our study, a case study design facilitated an in-depth analysis of the phenomena of regulated midwifery in Manitoba. First, we examined the number and distribution of midwives in the province over time; the results of this quantitative component have been described elsewhere [22]. In this paper, we describe the qualitative component of the case study. Qualitative description was useful in our study since the goal was to gain straightforward (low-inference) information to specific questions regarding issues that have impacted the implementation of regulated midwifery in Manitoba [26, 27].

**Fig. 1:**
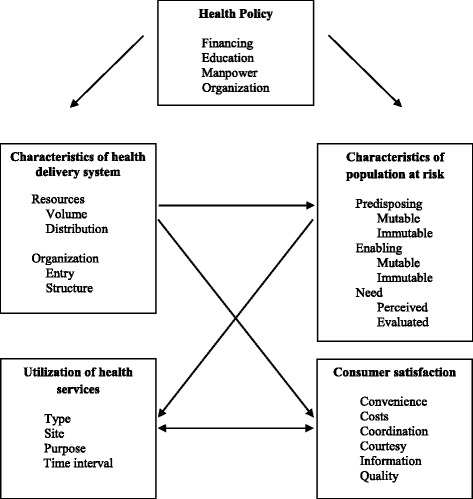
A Framework for the Study of Access. From “A Framework for the Study of Access to Medical Care”, by L.Al Aday and R. Anderson, 1974, *Health Services Research, 9*, P. 212. Copyright 1974 by Wiley. Reprinted with permission [36]

**Fig. 2:**
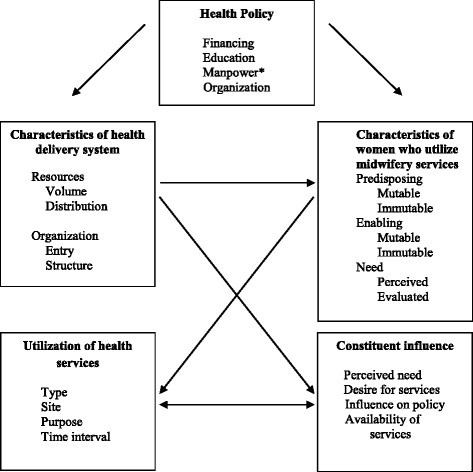
A Framework for the Study of Access (Adapted). Adapted from “A Framework for the Study of Access to Medical Care”, by L.A. Aday and R. Anderson, 1974, *Health Services Research, 9*, p. 212. Copyright 1974 by Wiley. Adapted with permission [36]. *The preferred language would be human resources versus manpower

The Behavioral Model for Health Services Use was used to conceptualize factors related to the utilization of midwifery services, such as health policy objectives and various characteristics of the health care system [28] (Fig. 1). The model has evolved to identify factors that facilitate or create barriers to the utilization of health services [29]. This framework was adapted to provide an organizational framework to conceptualize relevant variables specific to the utilization of midwifery services in Manitoba [28] (Fig. 2, adapted). In this model, health policy is seen as an important factor of how a population uses these services. The resources related to health policy are finance, education, manpower, and organization. The qualitative analysis assessed these issues in relation to the implementation and utilization of midwifery services. Perceived need for midwifery services was defined by key informants’ perceptions of women’s need for midwifery care. Other aspects of the model were explicated in relation to quantitative results reported in a different manuscript [22].

In addition, a feminist perspective was used to understand how the struggles and successes of the midwifery profession reflect a woman’s position in the health care system in Manitoba. While the Behavioral Model for Health Services Use framework was primarily used to guide this case study, consideration was given to how feminist epistemologies inform the interpretation of the results [30]. For example, the principal investigator was interested in understanding how the structures within the health care system and the influences of power within this system had impacted the utilization of midwifery services over time.

### Setting

Manitoba is a mid-western Canadian province with a population of just over 1 million people. The majority of the population resides in the urban areas (72 %), whereas the vast rural areas are sparsely populated (28 %) [31]. Winnipeg is the capital city and is the most densely populated metropolitan area (population of 730,018) in Manitoba. Health services are publicly funded and administered through five Regional Health Authorities (RHAs) [32]. The more recent birth estimates for Manitoba have ranged from 15,952 in 2009/10 to 16,237 in 2013/14 [33].

### Document data collection

All documents considered for this case study were publicly available and no permission was required to access the documents. The documents included evaluations, proposals, reports, archived documents, public announcements (multi-media sources), news releases, and internal records. All documents were historical in nature and gave insight regarding the following; evaluations of the midwifery program, previous proposals written to support the program, minutes from meetings which documented progress or lack thereof, and public announcements related to funding and other initiatives to support the profession. The various documents were organized according to the type and source of the document (Table 2). The criteria used to select document sources was that the information contained in the document was considered relevant to the research questions. The strategy for document analysis included review and examination of the research questions in relation to the document objectives [34]. The contents of the documents were then interpreted according to their objectives and if they validated and enhanced other sources of evidence.

**Table 2:** Documents from the public domain reviewed

Public Announcements (multi-media sources)/News Releases
1. (Periodical) Midwifery to become autonomous in Manitoba (1997) *Medical Post* 2. (Periodical) Legalizing midwifery has been a hard labor(1997) *Medical Post* 3. (Periodical) MB spends $1 M on midwifery before legalizing its practice: Service to be paid for (1999) *National Post* 4. (Newspaper) University eyes new BMW (1999) Canadian Press 5. (Newspaper) Growing pains dog midwifery in province (2001) 6. (News Release) (2004) First Aboriginal Midwifery Education Program to be established in Manitoba. 7. (Public Announcement) (2006) Health Canada. Aboriginal Midwifery Education Program 8. (Newspaper) Manitoba Health sat on midwife report (2008) *Winnipeg Free Press* 9. (Web) University College of the North (Programs Approved by COPSE in 2009/10) Program Expansion announcement 10. (News Release) (2009) Ministers Announce Expansion of Midwifery Training Program. 11. (News Release) (2010) New birth centre to be developed in south Winnipeg 12. (Newspaper) Birthing Centre disappoints (2012) *Winnipeg Free Press* 13. (Newspaper) Few born at $3.5-million centre (2012) *Winnipeg Free Press* 14. (Newspaper) Winnipeg’s new birthing centre ‘a colossal failure’ (2012) *Winnipeg Sun* 15. (Newspaper)Manitoba mom-to-be miffed she can’t use empty birthing centre (2012) *Winnipeg Sun*
Internal Records
1. Resolution from Manitoba Keewatinowi Okimakanak Inc. RE: Midwifery Services to Aboriginal women (2002)

### Interview data collection

#### Sampling

Key informants were purposively selected based on assumptions of who would yield the most thorough information in relation to the case study research questions [35]. Operational criteria for participant selection were defined by selecting representatives from the following categories: professional bodies, implementation groups, midwives (both practicing and non-practicing), health professionals and relevant personnel who collaborate with the midwifery profession, and provincial representatives.

The key informants were sent a confidential letter by email via the University of Manitoba server, to explain the purpose of the study with an invitation to participate. Of the 29 identified key informants from the defined categories, a total of 24 key informants agreed to participate in the study. Key informants were recruited from the following categories: Professional bodies (Manitoba Association of Midwives [MAM], College of Midwives of Manitoba [CMM]) (*n* = 4); Implementation groups (Working Group, Midwifery Implementation Council [MIC]) (*n* = 5); Manitoba Midwives (practicing and non-practicing) (*n* = 4); Health professionals and relevant personnel who collaborate with the midwifery profession (Medical directors, Nursing directors, Program Coordinators/Instructors of Midwifery education programs, Program Coordinators of the Midwifery Assessment Program, obstetricians and family practice physicians) (*n* = 7); and Provincial representatives (Manitoba Health, Regional Health Authorities [RHAs]) (*n* = 4).

#### Field techniques

The interviews took place from June 2012 to November 2012. Twenty-two of the interviews were done face-to-face and two of the interviews were completed by Skype (audio) due to the remote location of the key informants. Prior to the start of each interview, the key informants signed an informed consent form. Twenty-two of the interviews were tape-recorded by a digital recorder, and field notes were completed by the principal investigator immediately after leaving each interview. Two of the interviews were recorded with Callnote on Skype.

A semi-structured interview guide was used (Appendix A), based on three pre-defined topics (facilitators, barriers, and future strategies and recommendations) and guided by the adapted Behavioral Model for Health Services Use (Fig. 2) [29, 36]. Integral to case study design is the preparation of the researcher prior to initiating the study [25]. The three pre-defined topics came from the principal investigator’s preliminary work and history with the midwifery community. The principal investigator had done preliminary document analysis and casual interviews with key stakeholders on the policy framework of midwifery practice and the history of the midwifery education programs in Manitoba. Moreover, the principal investigator had personally been involved in Manitoba midwifery for 5 years in the capacity of a registered midwife and instructor for one of the midwifery education programs.

The Webster’s New World Dictionary (1991) defines barriers related to progress as, “anything that holds apart, separates, or hinders” [37]. In this study barriers are defined as factors which hinder the implementation or utilization of midwifery services in Manitoba. The word *facilitate* is defined as, “to make easy or easier” [37]. These factors can be grouped into categories of health policy, characteristics of the health delivery system, characteristics of women who access midwifery services, utilization of health services, and constituent influence as conceptualized in the Behavioral Model of Health Services Use [29]. Future strategies and recommendations was a preconceived topic that aimed to identify ideas within the results that had been recommended to move the profession forward.

### Data analysis procedure

All documents and transcripts of the interviews were analyzed using content analysis [38]. The basic steps of abstraction of data, creating codes, sub-themes, and themes [38] were employed to analyze the qualitative data from the document review and in-depth interviews. Data were initially coded by hand and then categorized in NVivo 10, and sub-themes were created. Overarching themes and sub-themes evolved in relation to each of the three broad topics.

### Ethics

Particular consideration was given to protect the anonymity of the research participants. The study was approved by the University of Manitoba Health Research Ethics Board (H2012:116) on April 27, 2012 and the approval was renewed annually.

## Results

Three overarching topic areas of facilitators, barriers, and strategies/future recommendations were pre-defined for the document and interview analysis. The key themes and sub-themes will be discussed below as they relate to the pre-defined topic areas.

### Facilitators

Participants commonly highlighted a strong feminist movement and key implementation strategies as positive influences on the profession. Many initial strategies were successful at facilitating growth in the midwifery program, which at least in part grounded the program so it could continue to make incremental progress.

***Theme One: Feminist movement:****constituent influence*

This case study used a feminist perspective to underpin the discussion of the findings, in order to bring awareness to factors concerning the utilization of regulated midwifery services that have been buried within structures of power. Subsequently, the goal was to find explicit meaning in how these factors could explain the position of women in the health care system. Midwifery was influenced by the feminist movement, in that women in the province actively advocated to make midwifery available as a choice for women.

Participants credited constituent influence as having a positive impact on the implementation process. Constituent influence was defined as any community member who had influenced the midwifery movement, which includes the consumer of the service. The majority of participants credited constituent influence as having a positive and significant impact on the implementation process. An initial core group of women cultivated midwifery in Manitoba, thereby bringing attention to the demand for the services, as mentioned by one person from the implementation groups:

*But certainly there was a an articulate and well-organized group of women who understood midwifery, believed in it, and fought for it for years and years in Manitoba, as there is with every other women’s issue in Manitoba…*

Overall, participants believed women’s strong desire for choice of birthplace and maternity care providers was the impetus to regulate midwifery in Manitoba. A participant from the professional bodies noted how supporting women’s choice was in essence the core impetus in fighting for the viability of the profession: “*Midwifery is really a movement about bringing women choice…Because without midwifery these [hospital] policies that are being written probably wouldn’t have ‘choice’ written anywhere*”.

Constituent influence was also highlighted as having an impact on the development of the midwifery-led Winnipeg Birth Centre that opened on October 31, 2011. As one participant commented:

*I think the Birth Centre was driven by consumers…it wasn’t just because midwives wanted a fancy place to work. Consumers came together and said, “We want a choice,”…“We want something that’s not a hospital.”*

***Theme Two: Key strategies.***

Several key strategies were fundamental in how the midwifery program was initially implemented in the province. The participants consistently noted milestones such as regulation, funding, integration of midwives into the health care system, and the opening of the Birth Centre as strategies that were successful components of implementation of midwifery. Funding and integration strategies also emerged in the document findings.

*Regulation.*

Participants commented on how implementation was successful, and the majority believed midwifery in the province had been fully implemented. Some participants shared mutual feelings that integration had worked. The development of transport and emergency protocols were given as examples. Most participants outlined various successes in the strategies to implement midwifery in Manitoba. For instance, a participant from the professional bodies commented on the fact that regulation was adequate since it included key components to support the profession:

*There was a very good human resource strategy…We’ve been well regulated…Midwives have been very equally a part of that regulation process. So as far as setting our standards, of being careful and aiming for safe practice, I think that regulation has done very well. Funded? Yes, we’ve been funded…The funding system is not a problem….We went through processes, we were assessed, the law was proclaimed, we were regulated, we had a regulatory body that made standards for us, the funding model was set as a salary model through the Regional Health Authorities…In those first years many people were happy with what was happening.*

*Funding.*

The implementation of midwifery as a funded service in the province was recognized as a key factor in facilitating access to these services. Manitoba had publicly committed to funding the service, which implied it would be an insured health service under the Manitoba Health Services Insurance Plan [18, 39]. In addition, midwives would be publicly funded, therefore would be salaried employees of the Regional Health Authorities (RHAs) in Manitoba [40]. An original member of one of the implementation groups commented on the successes leading up to the proclamation of regulation in 2000:

*We…and our supporters persuaded the government not to pass the Act until there were midwives ready to practice… We had a small core of midwives meeting the competency list that we had ratified. They were employed [by the RHAs]. It was going to be a funded service so women would not be out of pocket.*

Furthermore, from the time of regulation, a consistent commitment to funded midwifery positions by the government was noted in the documents. For example, Manitoba Health incrementally added new midwifery positions over time. From 2001/02 to 2009/10, an increase of 18.5 funded positions was reported in addition to the original 26 funded positions [41, 42].

*Integration.*

Participants commented that aspects of the integration strategy were successful. Integration was markedly fostered by undertakings such as the employment model, co-location of midwives with physicians, and collaborative work with the government and other health professionals. The employment model, whereby midwives were salaried employees within the RHAs, was consistently highlighted as an important and successful integration strategy, as explained by one implementation group participant:

*You had to force the integration, and we were a bit concerned about the fact that integration hadn’t been optimum…we wanted to ensure integration…The employment model has worked in that regard, in that…we’ve got midwives in different clinics…At least they’re there… and the doctors know they’re there. They can’t avoid them.*

*Implementation of the Birth Centre.*

In spite of some of the controversy of having a Birth Centre in Manitoba, some participants felt it demonstrated the government’s commitment to midwifery. Furthermore, it was one more avenue to increase the number of positions for midwives in Winnipeg. As one participant from a professional body noted:

*Now there’s been a huge influx of new positions being filled in Winnipeg. Part of that is because the Birth Centre, and it’s attracting attention, and new grads are thinking what a great place to work, and they’ve done a bit of recruitment, I think, or maybe it’s word of mouth recruitment because a few midwives have come here and, you know, from Ontario programs.*

In summary, key strategies, such as the profession achieving self-regulation, having public funding for midwifery positions, and establishing successful mechanisms for integration, all have demonstrated the public’s interest, support, and motivation to establish midwifery as a new profession within the larger maternity care system. Additionally, the constituent influence played an important role in garnering the government’s attention to implement the service. Finally, more recent endeavors, such as the opening of the new Birth Centre in Winnipeg, demonstrated the government’s commitment to women’s health care services in the province.

### Barriers

Four themes emerged under the topic of barriers. The first theme was related to conflict and power. The second and most prominent theme was education. The third theme was perceptions of the profession, and finally the last theme that emerged was a precarious profession.

***Theme One: Conflict and power.***

The theme of conflict and power was supported by three sub-themes (the nature of the beast, tenuous leadership within the profession, and turf and power) that illustrated aspects of conflict and power. These aspects of conflict and power have had a significant impact on how midwifery services have been used or made available within the broader health care system in Manitoba. Participants expressed that factors related to policy and political will were significant barriers to growing the profession.

*The nature of the beast.*

The nature of the beast refers to the power of policy and conflict in the context of government structures. For example, participants commonly identified policy and political will within structures of power such as the Regional Health Authorities (RHAs) as influential. The structure of the RHAs has had significant implications regarding how midwifery services are accessed and how a midwife practices. It appeared that fiscal restraints and the priority of services in the provincial budget were significant barriers to the expansion of midwifery services across the province. One representative of an implementation group commented on political will:

*Health care costs are escalating out of control and midwifery is seen as extremely expensive. Midwives are seen as expensive practitioners…It looks like a very Cadillac service…there’s huge demands on health ministers and there’s only so much money to go around. So I think it’s partly money and political will, and those two things are hand in glove.*

Another common barrier that arose was the disposition of a midwife as an employee of the government. A common concern among participants was that midwives ultimately struggle with being an “employee” of the health care system as opposed to being a fee-for-service practitioner. Participants often described how there were too many levels of management over the midwifery program, and this resulted in disempowering the midwife. One midwife commented:

*I feel like there are a lot of bureaucrats, like we have a lot of higher up, a lot of admin. In our program…we’ve got a clinical midwifery specialist, we’ve got a manager, we’ve got a primary care manager, we’ve got a birth center manager, we’ve got a manager at each clinic… where there’s this perceived thing that is being looked after but it actually isn’t*…

Other issues in the document analysis that aligned with the theme of power and conflict related to the hierarchy of management over the midwifery profession. Initially, the employment model was chosen as a way to facilitate integration into the healthcare system. The midwives, however, have felt constrained by the bureaucracy of the system in which they work. The Winnipeg Regional Health Authority (WRHA) Midwifery Program External Review document (2008) [43] highlighted problems regarding the management and roles of midwifery by revealing that medical doctors had “minimal understanding” of the regulatory structure for midwives in Manitoba. One significant issue was that midwives were generally over-evaluated by structures of management beyond their regulatory body.

Furthermore, it was also documented in 2008 that midwives were managed by four different managers, none of whom was from the midwifery profession [43]. The multiple levels of management created unnecessary bureaucracy, which made executing administrative tasks, such as reimbursement of expenditures and hiring, inefficient and complicated. Moreover, the constant change in management meant that those managing midwifery continued to have knowledge deficits about the Manitoba midwifery employment and practice model, which fed the tensions between midwives and management.

*Tenuous leadership within the profession.*

Tenuous leadership within the profession emerged as the second sub-theme under conflict and power. While there has been some midwifery representation at a government level, lack of political advocacy remains a barrier. Most participants felt political advocacy was crucial in maintaining power or a voice in the issue at hand. Unfortunately, many recognized childbearing only impacts people during a specific window of time in life and then they move on. A provincial representative explained how challenging it was to maintain political advocacy with the midwifery movement:

*It’s such a snapshot in the time of your life and then you’re done and you’re raising your kids and you’re moving on…The lobby…the faces are always changing because …once your youngest is three, four, five, you’re not focused on that [need/desire for midwifery care].*

*Turf and power.*

Turf and power was the third sub-theme that evolved under the conflict and power theme related to issues regarding professional conflicts. Some conflicts stemmed from turf wars among midwives and physicians. As one health professional stated, “…*We needed to put the ground rules first about who does what and where and when…When midwifery started, the family physicians felt as if we [obstetricians] were moving them out of the way*”.

It was interesting to note that while most participants felt midwifery experienced the greatest degree of resistance from other health professionals, at the same time, midwives created resistance against others in the health care system. One health professional shared the experience with midwives: *But there’s still in some of the midwives prejudice against us [physicians]…So there’s that element in midwifery that does not respect our ability to deliver a low-risk pregnancy.*

Inter-professional conflict also emerged early on during the process to achieve regulation. The document analysis revealed that in 1999 a discussion about the possibility of midwifery education in the context of the University of Manitoba was happening between midwifery stakeholders and the University. Both parties were concerned about how midwifery would be integrated into this educational institution. Furthermore, the midwifery community had expressed concerns that the integration of midwifery into the Faculty of Nursing would “jeopardize the integrity and autonomy of the profession of midwifery” [44]. Once again, the midwifery community was divided and did not fully endorse the proposal but was not in a position of power to influence the direction of education at that time.

Intra-professional conflict was also a common concern expressed as a barrier that relates to conflict and power within the midwifery profession itself. In the beginning, nurse-midwives experienced tension with direct entry (non-nurse) midwives. The differences in philosophy of care between various midwives appears to have created a barrier. One noteworthy example of why these differences exist was portrayed by one person from the implementation groups: *“There are still philosophical differences, but you even see those between two different nurse midwives…who have nursing backgrounds, and two different midwives who came through a different route”*.

Another common feeling expressed by participants was that midwives can be their own “worst enemies.” A participant from the implementation groups noted how intra-professional conflict can be detrimental to the profession:

*I think midwives can be their own worst enemy or they can make, they can elevate their own profession to a level where they are seen on equal footing with other professionals and well-respected and integrated…. you need to figure out how to work with everybody … I get the different philosophy and… and women’s choice. But I think that’s how they’re their own worst enemy because those kinds of interpersonal things suck the energy out of people.*

In summary, structures of power, issues of political advocacy, and inter/intra-professional conflicts were threads to the theme of conflict and power. Historically and currently, midwives have been subjected to structures of power, including those of other professions. However, midwives, at least in part, have some responsibility in the way they have positioned themselves as professionals. Furthermore, these tensions between midwives impedes the ability of the profession to affect political action.

***Theme Two: Education.***

Education was the most prominent theme that emerged under barriers in both the document and the interview data. The two sub-themes that emerged demonstrated challenges within the system: types of training, and lack of a productive midwifery education in Manitoba.

*Types of midwifery training and assessment.*

The first sub-theme focused on how the different types of midwifery training and assessment have created barriers. Participants brought attention to the inherent challenges related to the various types of training Manitoba midwives have brought to the table. Midwives often come from very different educational backgrounds. The identified barriers were attributed to the foreign trained midwives’ lack of knowledge regarding the model of practice of midwifery in Manitoba, the lack of assimilation of their own skills within Manitoba midwifery standards of practice, language barriers, and other issues. One health professional commented on how variances in how the midwife is trained have created “angst” amongst the physicians:

*For example, so many of the midwives come from out of the country.… That’s part of the angst of the physicians they had at the very beginning, ‘every midwife coming from a different background was doing different things. Their ideas, their way of pain control, their ideas of pushing, their ideas of delivering, their ideas of episiotomies, of stitching, etc. Everybody was doing different things, depending on their training.*

*Lack of a productive midwifery education program in Manitoba.*

Participants consistently noted that the failure to develop a productive midwifery education program was one of the most profound barriers impacting the growth and sustainability of the profession in the province. Many stakeholders felt that not having a solid educational strategy embedded in the implementation process was a significant flaw within the process. A provincial representative commented on how midwifery education should have happened earlier after implementation, and said: *It was a big gap when we did not have an education program in Manitoba in terms of growing the profession, developing an understanding…You know, it was a real gap in terms of credibility of the profession when we didn’t have an education program.*

Moreover, many stakeholders felt discouraged that the original proposal for a bachelor of midwifery education program at the University of Manitoba never came to fruition.

Similar concerns were noted in the document review regarding the overall trajectory of midwifery education in Manitoba. When the proposal for a midwifery program at the University of Manitoba was being developed prior to regulation, support was expressed from both the provincial government and the University of Manitoba. The Council on Post-Secondary Education (COPSE) also granted their approval to proceed with full development of the midwifery proposal for the School of Midwifery [44]. However, in 2001 when the College of Midwives of Manitoba (CMM) met with the government, they were told that no new plans for funding midwifery positions were on the horizon [45]. As a result, the CMM was told the resubmission of the midwifery education proposal would not likely get funded [46]. Understandably, midwifery stakeholders in the project were frustrated or discouraged by the sudden lack of political will. All of the time, money and effort expended to develop the midwifery education proposal did not come to fruition.

Shortly after the proposal for midwifery education at the University of Manitoba was deferred, the provincial government announced the first Aboriginal midwifery education program in Manitoba [47], and provided funding to the University College of the North (UCN) to offer the program, known as the Kanácí Otinawáwasowin Baccalaureate Program [KOBP] [48]. Many participants outlined their concerns regarding the choice of UCN to house the KOBP in northern Manitoba. UCN was believed to lack the capacity to deliver a successful and sustainable midwifery program in Manitoba. One health professional described the issues regarding UCN’s capacity to deliver the program as follows:

*But they didn’t have people there physically who were able to develop the program, so I think that’s partly why it ended up being developed outside the auspices of the institution….There was a dithering… on actually getting the funding forwarded [to UCN] … to the point that they actually had half the length of time, 18 months, to actually do all the work for a 3-year program…When the program was launched in’06 we had two instructors, one in each location…They were attempting to be practicing midwives full-time, as well as full-time instructors. It just fell apart. They [instructors] didn’t have the support they needed from UCN, from communities, from the dean, from anybody to actually accomplish those roles.*

The KOBP program started in 2006 but had not produced any graduates as of 2010 [49]. In 2010, UCN announced a new intake for a “southern cohort” of the program, re-located the program to Winnipeg, called it a Bachelor of Midwifery Program (BMP), and stated the midwifery program would likely not be “delivered in its original design” [50]. As of 2014, the BMP recently yielded ten midwifery graduates. Challenges of the educational programs identified in the documents and by participants in the study were difficulties with the recruitment of instructors, lack of clinical sites and preceptors, especially in northern parts of the province, and lack of overall capacity of the UCN to manage the program. Moreover, the feedback given to UCN about the original KOBP was not acted upon prior to pushing forward with a southern program [50].

The theme related to education draws attention to the inherent problems of how midwives are trained and the lack of leadership and infrastructure needed to launch a successful midwifery education program in the province. The diversity of the midwives’ backgrounds could help explain how other professionals often misunderstand the role of the professional midwife. Integral to any sustainable health profession is a sound and credible educational program. Consequently, without midwifery student integration into the academic institutions that educate other health professionals, midwifery remained a silo and poorly understood.

***Theme Three: Perceptions of the profession.***

The third theme comprised barriers related to perceptions of the profession. Within the perceptions of the profession, stereotypes and misunderstanding of the professional scope of practice evolved as problematic for the profession in gaining credibility.

*Stereotypes.*

Many participants believed the society in which we live perceived the midwifery profession as antiquated. The attribution of these common stereotypes to midwifery appeared to have weakened the overall image of the profession. Ultimately, these stereotypes have impacted midwives’ status negatively in other professional circles. Consequently, the issue of women’s choice seemed secondary because midwives did not appear to be *valued* as a legitimate health care provider. The public’s skepticism and stereotypical views of midwives was notable in the interviews as discussed by a health professional and a provincial representative. One of them explained that “*I would have to say that there was probably a lot of skepticism on the part of the public because they didn’t know. Everything they knew about midwifery perhaps came from a book, from folklore, from poor reports, etc.*”

The second participant expressed:

*I think in the case of midwifery…some of the issues around the implementation of midwifery initially were still the stereotypical views held by some lawyers, bureaucrats, physicians, particularly men in those professions, you know, who still saw midwives as, you know, non-professional, you know, hippy, dippy, granola belt, you know, all of those stereotypes about midwifery. They didn’t see it as a profession. They … didn’t get it in terms of the fact that women wanted choices*.

*Misunderstanding of the professional scope of practice.*

A similar issue regarding perceptions of the profession was in relation to the misunderstanding of midwives’ professional scope of practice. Participants remarked that the media has played a role in portraying midwifery as an unsafe profession. Two participants from the implementation groups and the provincial representatives voiced that unfortunately when a bad outcome happens with midwifery care, the media garners the public’s attention. As one provincial representative stated, “*It [bad outcome by midwife] gets steamrolled [by the media] into something that is untrue…*” The other comment made was, “*Whenever there’s a problem [bad outcome by midwife], it really gets the press*”. Perceived misunderstandings regarding safety and the skill set and training of midwives were raised by the participants. One participant from the implementation groups gave details of how midwives’ skill sets are called into question and misunderstood in the health care system in which they work:

*So early on, I think it was lack of understanding that midwifery was a regulated profession, that they actually had training and experience and expertise to offer. There was controversy around home births and the sector of health care system believing that was an unsafe practice… And home birth was always, before regulation, was seen as so outside of the norm of the medical system, that people were uncomfortable…You know, we ran into situations early on where they didn’t realize that midwives actually brought equipment and oxygen and, like, so we had sessions, midwives bring their birth bag, open them up, and say, “This is what we bring to a home birth,” …They [physicians]were shocked that midwives actually had a bag of equipment…I think they thought it was truly no intervention, like, a midwife was just sitting there watching this natural thing happen.*

The opening of the Birth Centre in Winnipeg provoked strong emotions about safety and the cost-effectiveness of this type of maternity care in the context of a multi-million dollar maternity care facility. One health professional felt that “*there was lots of money spent on this thing and now it’s [Birth Centre] underutilized,*” and compared it to the medical model of maternity care: “*[The Birth Centre does] 46 deliveries in 6 months. We do 46 here [hospital] in 2 days”*. Safety issues were also associated with the Birth Centre environment, similar to the issues of the homebirth setting.

The stereotypes and misunderstanding of the professional scope of midwives have fostered a culture of uncertainty within society regarding aspects of the model of midwifery care. Understandably, the way midwives have been perceived contributes to the continual battle to establish credibility within society and the professional domain.

***Theme Four: A precarious profession***

The final theme that emerged was in relation to precariousness of the profession. Various aspects of the profession are linked to barriers which have impacted how midwifery services are used or made available within the broader health care system in Manitoba. One participant alluded to the midwifery profession as “*being ‘precarious’ because of all the instability and lack of growth in the profession*.”

*Lack of capacity.*

The lack of capacity addresses several specific issues that have impacted why the midwifery profession has struggled to grow. Issues related to recruitment and retention have influenced the lack of capacity. One health professional commented: “*Midwives were leaving the province because they are realizing it is not a sustainable profession*”. According to participants, recruitment and retention initiatives had not been addressed and this created a barrier to increasing access to the services. There had been no coordinated provincial plan or strategy. One health professional identified a specific issue related to the problem: “*I think having more provincial recruitment [of midwives]; right now every RHA is on their own trying to recruit and so it’s not very effective*”.

Another common issue related to lack of capacity within the profession cited barriers within the current model of practice and employment. The model of practice was commonly perceived as a barrier which has impacted the capacity of the overall profession to provide greater access to midwifery services. The model sets a low limit for a midwife’s caseload. Generally, a full-time midwife will take on approximately three to four clients per month, while a part-time midwife will take on one to two clients per month. One participant from the implementation groups commented:

*I haven’t seen any reason to not have the kind of model of care that we have in this province with 40 primary cases for those who are working full time, but I just don’t think it’s sustainable when there’s no new growth of the midwifery so that people can take a break, so midwives can take a proper holiday and have locums fill in for them, and I just think it leads to burnout. You just can’t keep relying on the same people for 15 years to do the same work all the time*.

As well as the model of practice, the model of employment was associated with barriers which impacted the capacity of the midwifery program to expand services. Participants perceived many initial benefits to the salaried employment model. However, some participants began to view the salaried model as a barrier that decreased the capacity of the profession to grow, inhibiting access to the services. Barriers have been created by the circumstances that the employment model appears to perpetuate, such as lack of accountability among midwives, lack of choice for midwives with regards to employment setting, and limited caseloads per midwife with no system of accountability with regards to the number of caseloads. One midwife divulged how the salaried employment model had possibly contributed to decreasing women’s access to midwifery services:

*So I see that as a barrier because we are not servicing the number of women we could be… that is a huge problem right now in Manitoba and, as we’ve seen, there’s just not as much accountability with regards to the numbers and caseload number… there’s no manager saying, “Hey you’re full time, how come you don’t have 40 people in your care?”…And I think people are abusing the system…I just don’t think if somebody was to do a cost analysis, that just would reflect very poorly on the employee model.*

The participants also expressed concern with the lack of capacity in the profession in relation to ensuring midwifery access in rural and remote areas. Two participants commented that the efforts to integrate services in the north were met with resistance by certain communities. One provincial representative stated, “*This resistance came from the First Nations people’s fear of going backward. Thus, midwifery care was perceived as second-hand care*”. In general, the majority of the participants felt the province had fallen short of providing access to these underserved areas, predominantly in the north. One participant, from professional bodies and health professionals, elaborated on the importance of maintaining equity and access as foundational to the nature of midwifery services, especially for northern communities.

*I maintain this sort of passion for this issue of equity and access, and I think that is a really key piece that can’t be forgot. I don’t think it has. I think there are pockets where it is, but the fact that we really don’t have, there’s … like, three midwives that work in the north, and one’s on maternity leave, and one’s retired, and the other one can’t do birth…I’m talking about this other aspect – northern midwifery, rural midwifery, newcomers, remote people in the remote, adolescents, Aboriginals, all those pieces. That was the focus of midwifery in Manitoba…I think people will forget about equity and access priorities because they will be so drawn into the moment of today, which is we’re overwhelmed, or there’s too many, no one gets any care, or this and that but what were we really built on?*

Finally, lack of capacity in the midwifery profession was linked to midwifery being a small, vulnerable profession. Many participants mentioned that midwives have too many roles to fill. Some felt this had a domino effect on the capacity within the profession to fill preceptor and instructor roles with qualified midwives. Overall, many participants acknowledged a small body of midwives was a substantial barrier to increasing capacity because people are burned out with the current workload. One person from the professional bodies indicated there are not enough people to get the work done, especially with the efforts to expand the overall profession:

*As you continue on without the education piece sufficiently in place and, again, the association piece sufficiently in place, you start to see the impact on the regulation side …when you don’t have enough people to do all of the work that the College needs to do. You don’t have enough people to support the new practitioners. You don’t have enough people to support the education program and that sort of thing.*

In summary, access to midwifery services is compromised by multiple factors related to the profession’s lack of capacity to meet the demands. Consequently, the profession is constrained by limitations of the model of care/employment, lack of a clear cohesive provincial plan for recruiting and retaining midwives, and finally midwives committed to too many roles such as clinical practice, maintaining the College of Midwives, education, committee work, and implementation work. The profession remains in a precarious and vulnerable state due to increased demands without a sound education program and a human resource plan for a consistent increase and retention within the workforce.

### Future strategies and recommendations

Key stakeholders were given the opportunity to discuss at length what they deemed as critical endeavors for the future direction of midwifery in Manitoba. Therefore, the third pre-defined topic aimed to understand what future strategies and recommendations should be examined. Four themes related to future strategies and recommendations arose from interviews with the key stakeholders: avenues for midwifery education, refocusing management strategies, evaluation of the midwifery program, and the need for more research.

***Theme One: Ensure avenues for midwifery education.***

The first theme, to ensure avenues for midwifery education, was almost unanimously suggested by participants. For example, gap training was suggested as needing to be addressed and embedded into the midwifery education program. Prior learning assessment (PLEA) was seen as an essential strategy to target trained professionals, who had previous midwifery training, but just needed assessment and gap training to be eligible for registration as a midwife in Manitoba. Gap training was recognized as a need and an important tactic to increase the number of licensed practicing midwives in the province. One health professional commented:

*The strategy of trying to … look at different programs such as the PLEA to try and get more practicing midwives in the province was probably initially a good one. It just was incomplete because of the gap-filling aspect…I think we’ve got the people who are already trained partly. Can we not find ways to help those people bring up their education to the level that’s equivalent in whatever fashion we do that?*

Likewise, some participants strongly suggested it was critical not only to implement a sustainable midwifery education program, but that a school of midwifery should exist in the context of a faculty of health professions. Two participants from the health professional group suggested the educational program also needed to match the demand for midwifery-funded positions. One person stated the importance of “…*government funds matching educational positions*” so that midwives who graduate can go directly into positions. Another participant pointed out, “…*if you ever start training people that can’t find work in health care, that’s money that you should not be spending*”. Overall, participants deemed “growing our own” as critical to the sustainability and full integration of the profession.

Finally, many participants felt it was critical to continue to focus on midwifery training in the northern communities. One participant commented on the need to refocus the commitment in the north and rural communities:

*I am still very dedicated to the idea of training…Aboriginal midwives in the north. I think we need to address the issue of women having to…go so far away from home. Maybe they can’t birth in their community, but wouldn’t it be nice if they could birth closer to their community? It seems to me like there’s a resignation that women will travel to Winnipeg …We have to make a much stronger effort to reach out to that population.*

***Theme Two: Refocus management strategies.***

The second theme encompassed policy issues related to management and accountability issues within the midwifery profession. Many participants felt more accountability and leadership was needed within the government, but also within the direct management of the midwifery profession.

*Government/RHAs.*

Several areas of concern surfaced during the interviews such as the need to refocus strategies within the government to expand and support midwifery in the province. Moreover, the midwifery work environment was consistently highlighted as needing attention, followed by action. Another topic mentioned was the need for a recruitment and retention strategy. Several participants also suggested that the RHAs should be mandated to implement midwifery programs. One provincial representative addressed many of these matters:

*I think we need to refocus…get those [midwifery] positions filled, work with regional health authorities, almost another kind of targeted piece of work with the regions to make sure that midwifery is not, you know, on the back burner in the regional health authorities. So education, some targeted work with the regional health authorities regarding, you know, active recruitment into those positions, additional positions, deployment into every region in the province and then, as soon as we’ve got sufficient resources, doing some communication campaign.*

In 2010/11, a proposal was submitted to Manitoba Health by the CMM to request that they “take a lead on the coordination of a recruitment and retention strategy” [50]. However, there was no noted follow-up on this strategy.

The majority of participants commented there was a need for improved accountability frameworks within government related to transparency regarding evaluation results and subsequent strategies for program planning and expansion. In June 2011, Manitoba Health conducted an evaluation of midwifery services in the province. In spite of the government’s attempts to manage and evaluate the midwifery program, there has been a sentiment of dissatisfaction with the outcome of these initiatives and these reports have not been made public. This was expressed by one midwife:

*I think communication of what the issues are and then having good forums for problem-solving would be helpful. So I think your uncensored Manitoba health survey of these issues, is very helpful because the survey that was done by the Manitoba Health group not that long ago was obviously catered to an agenda…*

*Work milieu.*

Some participants commented on the work environment as a factor contributing to burnout and high rates of attrition for the Manitoba midwives. According to one health professional:

*There needs to be other policies in place or protocols or guidelines in place that say, you know, okay, well, we’ve got this many midwives, so you’re on call for the next 48 h, and then the next midwife, like some kind of a rotational call system, where it actually prevents you from working 100 h a week.*

***Theme Three: Evaluation of overall midwifery program****.*

The third theme addressed the need to evaluate the overall midwifery practice, which encompassed the model of practice and the model of employment. The model of midwifery practice in Manitoba delineates 11 fundamental principles, as defined by the CMM [9]. The employment model in Manitoba is based on a salaried model. In Manitoba, the provincial government provides funding for midwifery positions, therefore most midwives are salaried employees of RHAs [51]. Participants voiced the need to evaluate the mandate for priority populations and home birth, as well as the need to evaluate the current model of employment.

*Evaluate model of practice.*

A critical examination of how priority populations are defined arose from the interviews. As one health professional reported, priority populations were not well defined; thus an evaluation of the current definition was warranted:

*I also think we need to better define those [priority populations], if we’re going to really be serious about trying to give priority to our priority populations…We haven’t defined them well provincially or regionally…This woman’s an immigrant but she’s lived in the country for 10 years, so really is she an immigrant? I would argue that, right?…Or is it a newcomer who to me is less than 3 years in the country of whatever, right?…I think it’s very subjective.*

*Evaluate model of employment.*

The stakeholders from the health professional group suggested an evaluation of the salaried employment model would be beneficial to help understand if it is still the most appropriate model.

Another participant among the health professionals stated the following:

*I think our province needs to change our payment model; it’s not just about the money… I’m saying I think we need to change the structure of the employee/employer relationship or structure because if we were more independent practitioners and we owned our own practices and we worked for …fee for service or per course of care, we would own it.*

Ongoing work related to the employment model was documented. In 2005/06, Manitoba Health [52] encouraged the College of Midwives of Manitoba to consider reevaluation of the employment model and that it might “not be sustainable.” A focus group was formed a year later, and funding was requested to do the research and evaluation of the employment model. No follow-up had been noted in the documents related to the requested proposal for the evaluation of the employment model.

***Theme Four: Research.***

Finally, a theme related to research was the fourth most emphasized strategy emerging from participant interviews. Participants articulated the need for more research in relation to the utilization/distribution of midwives and the initiatives of the midwifery profession in the province. Participants felt a more critical analysis was needed to understand funding issues with midwifery positions, which would help to inform the government more accurately about the facts.

Many participants voiced concern regarding aspects of Manitoba midwifery which had not been well articulated in formal research. One health professional outlined gaps with midwifery research in the province:

*I think they need to look at everything more globally. You know, do the sound work on how many women in Winnipeg actually would prefer midwifery care and not pie in the sky numbers and, and look at how to fill the needs outside Winnipeg…So there’s this almost religious fervor around midwifery, but not actually looking at the numbers and true need…And that’s harmed it I think.*

***Interviewer:****…What has it harmed…?*

*The rational distribution of midwifery resources in Manitoba…You’ve got to do it based on real good solid data, and you’ve got to do it in partnership, and each partner has to respect each other’s abilities.*

Various attempts to promote midwifery research and data tracking processes were noted in the documents. The College of Midwives of Manitoba (CMM) was concerned that the (then current) “distribution of midwifery services fails to promote equity” and were also concerned that no province-wide evaluation had been done. The Prairie Women’s Health Centre of Excellence (PWHCE) provided seed money for the research proposal to examine these very issues, however, the proposal was never funded. There was also documentation that CMM was working with Manitoba Health to “develop processes to track data through the midwifery discharge form” [53].

It appears that any future strategy must address a more successful mechanism for midwives to attain training and registration in the province. Other strategies need to target accountability within management structures where attention has been given to issues yet action has not been evident. Finally, evaluation and research go hand in hand to improve the delivery of the program. Without an effective documented evaluative strategy, it is hard to explore what specific research initiatives need consideration.

## Discussion

Due to the breadth of the study results, the key findings will be discussed in relation to the major characteristics of the health policy components of the Behavioral Model of Health Services Use framework developed by Aday and Andersen [36]. While many themes aligned with barriers, participants commonly highlighted successful implementation strategies such as funding and integration as positive influences on the profession. Other aspects of implementation, however, such as establishing a successful midwifery education program, remain a challenge.

The two most prominent themes from the topic of barriers informed by the Behavioral Model of Health Services Use were conflict and power, and education. The interrelation of health policy factors such as finance, education, manpower or human resources, and organization explains how midwifery health services were utilized.

### Health policy

The findings in our study were consistent with the literature from other provinces regarding the impact of policy initiatives related to the implementation of the midwifery profession. For example, Ontario, Alberta, and British Columbia initially fought for fundamental aspects of midwifery: midwifery regulation, education, integration into the health care system, and a publicly funded service [54–56]. As demonstrated in our findings, Manitoba’s policy goals appeared to establish fundamental aspects to fully implement midwifery services in the province. In June 2000, the Government of Manitoba followed through with the commitment to offer access to midwifery health services by proclaiming the Midwifery Act [57]. The Manitoba government had in essence met their initial policy goal. The enactment of this policy fulfilled the government’s commitment, set forth in the 1994 public announcement, to regulate midwifery. Manitoba appeared to have a sustainable midwifery implementation strategy, as evidenced by endeavors to publicly fund the service [39] ensure accessibility to services by the Aboriginal population [58], and fund a midwifery education program in the North [59]. However, the findings of this study suggest that these initial and fundamental steps to establish midwifery in the province have not been followed by effective implementation actions over the subsequent 14 years to ensure the growth and sustainability of the profession

Access to health services or, in this case, midwifery services is realized through utilization [29]. Aday and Andersen [36] suggest that access is more of a “political idea versus an operational idea,” which makes it hard to effectively evaluate programs. The Manitoba government made a political decision to implement midwifery. The policies to support the midwifery program, however, were not cohesive and lacked a strategy to effectively move the profession forward.

#### Finance

Finance or funding was one characteristic of health policy perceived as a facilitator that impacted the utilization of midwifery services in Manitoba. Funding for midwifery services has been a challenge in other provinces, where the government’s voice has reflected the rhetoric of women’s choice but its commitment has often been vague [56]. Alberta’s regulation of midwifery was different from regulation in British Columbia, Ontario, and Manitoba in that the government had taken no initiative to fund midwifery services in the public health care system [55].

In Manitoba, there had been a substantial amount of money committed to support midwifery services from 2001/02 to 2009/10 which gives the impression that women’s voices were being heard at a policy level. Many participants agreed the government’s commitment to fund the services contributed partly to the successful implementation of the services. Our study revealed that the government demonstrated financial commitment to midwifery services in ways such as an increased number of funded positions over time [20, 60–62].

In spite of the government’s commitment to fund midwifery services and education in Manitoba, participants noted that there has been a lack of accountability and few mechanisms for monitoring these financial decisions.

#### Education

In Canada, midwifery was founded on the three pillars of a strong midwifery profession as defined by the International Confederation of Midwives (ICM), which are education, regulation, and association [63]. A key element of the profession that has been missing in Manitoba is a midwifery education program that produced a steady flow of graduates to add to the number of midwives in the province.

In Manitoba, the profession of midwifery has made several attempts to uphold midwifery knowledge, thus preserving its traditional skills and philosophy of the model of care. This is evidenced by the successes of a self-regulated profession which has defined principles and processes for the midwifery model of care in Manitoba. The findings from our study highlight, however, how structures of power have fostered barriers for midwives to fully expand the profession. Furthermore, these barriers have created consistent tensions between the midwives and the system in which they work. Specifically, midwives have lost some control of their profession by not having a productive midwifery educational program. The lack of a productive educational program has influenced how the midwives have been professionally socialized into the health care system. Midwives in Manitoba continue to face battles to justify their professional status within the system. Their backgrounds are unknown to their health care colleagues, and the midwifery education students have not had a strong presence in the clinical setting.

Other provinces have demonstrated how a successful baccalaureate program is critical to the sustainability, growth, and credibility of the midwifery profession. For instance, Ontario and British Columbia have recognized that baccalaureate-level midwifery education was essential to increase the credibility of midwifery in the health care system. In spite of the controversy about how to educate the midwives, Ontario and British Columbia established a baccalaureate program instead of a community-based apprenticeship [54, 56, 64]. Baccalaureate education for midwives was also seen as important to the integration of midwifery in Quebec. Two studies from Quebec noted that a university degree for midwives was recommended [65, 66].

#### Resources (manpower) and organization

The resources and organization of the health care system are included in the Behavioral Model of Health Services Use [29, 36] and are considered attributes of health policy. Variables within these attributes can explain how resources and the organization of the midwifery profession have impacted the utilization of the services. Successful implementation strategies illustrate the resilience of the profession and the ability to remain viable. In spite of many barriers, the profession has used manpower-related and organizational efforts to develop foundational mechanisms such as standards, policies, guidelines, and bylaws [43].

Several key factors have acted as barriers resulting in the lack of professional capacity. One policy effect on the midwifery profession as it relates to the conflict and power theme was linked to the Regional Health Authorities’ management policies. The midwives have interacted with multiple layers of management within the Regional Health Authorities, and whenever their input was sought they felt their voice was not heard or considered. Both the documents and interviews revealed how midwives were overly evaluated and managed by their employers, the RHAs. The excessive management of the midwives was interpreted as lack of knowledge within the systems of the regulatory structure for midwives.

Some inter-professional challenges have stemmed from the differences between how the midwives and other health care professionals within the RHA health system have defined the principles of their models of practice. Similar struggles with maintaining the midwifery philosophy have created tensions between midwives and the health care system, and have contributed to inter-professional conflict in other jurisdictions. In an analysis of integration issues of midwifery in British Columbia the following question was of primary concern: “How can midwives gain the support of dominant players in the health care system without sacrificing the crucial elements of independent practice?” [67]. This question articulates the effects of the struggle of midwifery professionals for autonomy not only in British Columbia but also in Manitoba, as found in our study. Kornelsen noted that demanding autonomy creates alienation from the professions of medicine and nursing, yet not protecting the philosophy of midwifery places the model of care at risk of being altered. Ultimately, the effect of the struggle for autonomy explains, at least in part, why the inter-professional relationships of medicine, nursing, and midwifery have been strained [67].

Unrelated to the conceptual model, our study found barriers related to inter and intra-professional conflicts. These findings are in line with the type of barriers encountered in other provinces. For instance, a longitudinal case study of midwifery’s professionalization in Alberta theorized that the lack of governmental support after legalization was related to two issues; inter- and intra- occupational conflict and the province’s influence on initiating, implementing, and then restraining midwifery’s professionalization [55].

In our study, participants discussed issues related to inter-professional and intra-professional conflicts. In Manitoba, the medical and nursing professions have demonstrated resistance to midwifery as a profession. From the outset of regulation, representatives of nursing have expressed negative connotations about the role of the midwife. A survey administered to practicing maternal-child nurses in Manitoba was completed to understand what they thought of the midwifery model [68]. One finding from the study was that 86 % of the respondents projected maternal morbidity and mortality would increase with the introduction of midwifery into the health care system [68]. In other provinces, such as British Columbia, Ontario, and Quebec, contentious issues between midwifery and the nursing and medical communities have always existed [23, 54, 55, 64, 65, 67, 69].

Alberta midwives demonstrated similar tensions from within the profession, specifically between non-nurse midwives and nurse midwives [55]. In our case study, the infighting in the early years after regulation was specific to non-nurse midwives and nurse midwives; however, in general, midwives were seen as their “own worst enemy.” A common sentiment noted among participants in our study was that “infighting” created a negative presence for midwifery, as well as an energy drain for the profession. Similarly, infighting among the midwives in Quebec created disunity within the profession, but more importantly portrayed the profession as disjointed in the eyes of the government [70]. In Manitoba a lingering tension exists within the profession that limits strength and cohesiveness to advocate for the profession with those who hold the power to make changes to ensure that midwifery is sustainable.

#### Findings unrelated to the framework

The framework was helpful for understanding health policy and how it affects the utilization of midwifery services. What the model does not address, however, are the specifics of the structures of power vis-à-vis the culture of health care personnel or the gender issues within the system and how they may impact access and utilization. In this study, noteworthy findings associated with barriers were related to the dynamics of gender and structures of power. Parallel struggles with the implementation of midwifery services across Canada have been fraught with challenges related to the autonomy of the profession, government push back, and the hegemonic medical discourse [55, 56, 64, 69–74].

Gender has historically influenced the organization of the midwifery profession. For example, one author suggests feminist ideologies were used in Canada to challenge medical claims that natural childbirth was unsafe [75].

In this Manitoba case study, participants voiced concerns about midwifery’s struggle to practice full autonomy as a profession related to structures of power such as medicine and the government. In Alberta, midwives also underwent a tremendous struggle to establish credibility and maintain autonomy within the health care system. The Alberta midwives’ lack of ability to grow the profession was related to the marginalization of the profession by medical dominance. Medical dominance appeared to inhibit full autonomy, and lack of funding was related to the right wing government’s “cost control policies” [55].

Within structures of power, gender was viewed as a barrier to accessing the provider type and choice of birthplace that women desired. Historically, physicians in Manitoba held the attitude that men with formal training were far superior to midwives and made childbirth safe. Secondly, doctors paid for their formal training and therefore believed their investment in medical school warranted a more lucrative pay for attending childbirth [76]. The concept of gender has been used to help understand health-seeking behavior in women as it associates power with gender [77]. Women can be viewed as powerless with regards to their health-seeking behavior if the outcome of their health in a patriarchal setting is viewed as beyond their control [77]. For example, in the context of this case study, a woman’s decision to utilize midwifery care is considered health-seeking behavior. Participants also verbalized that the small number of existing midwives impacted the profession’s overall capacity to grow due to the many demands on the individual midwife. Therefore a woman’s decision to access a midwifery provider and her choice of birthplace was beyond her control because of the limited midwifery resources available to women in the province. The territorial nature of maternity care providers fostered conflict between genders. Power plays were noted as a two-way problem. First, other health professionals were seen as intolerant of the midwifery profession. Likewise, health professionals often interpreted the midwife as not wanting to integrate into the mainstream health care system.

The scientific discourse of the medical model has somewhat shaped how midwifery policy has been implemented in relation to medicine’s concerns about the safety, competence, and expertise of maternity care providers. In this sense, midwifery has moved away from being centered on the woman’s reproductive rights towards the midwife as the primary decision-maker [78]. In Manitoba, these same concerns have created tensions with professional groups and structures of power, whereby midwives have had to justify their role as autonomous primary care providers, in spite of existing regulation. Although British Columbia and Ontario have had many successes, some communities within those provinces have experienced similar resistance, as noted in a case study from the medical community, which created barriers for access to midwifery services [72].

### Limitations of the study

This case study has limitations. The study relied on interviews as the primary source of data. The quality of the interviews was influenced by individual’s time constraints, recall bias, and personal philosophy and stance towards the midwifery profession. Many participants in this study communicated burnout from the profession, which may have influenced their responses. Occasionally, it was evident that participants had frustrations with a specific issue and had to be redirected during the interview process. Most participants were busy professionals. In particular, time with physicians was limited to less than an hour for the interview process. In examining various documents, the inconsistent reporting of data was often noted between sources in the document review. Finally, caution is needed in generalizing the results to other provinces or countries, because the case study focused on the implementation and utilization of midwifery in Manitoba. However, many of the themes arising from the interviews were consistent with findings from studies conducted in other Canadian provinces.

### Recommendations for future research

Several suggestions for future research arose from the results. One recommendation is to conduct an evaluation of the current employment model. The model of care needs to be carefully factored in throughout this analysis, while considering the existing literature related to cost analyses of midwifery care from across Canada [79–82]. Another recommendation relates to data tracking. From the inception of the midwifery program to 2012, Manitoba Health has not generated annual statistical reports for midwifery services (J. Watt, personal communication, June 20, 2012). There needs to be a mechanism in place that streamlines the tracking of data related to the number of midwives, the reasons for attrition, and other relevant information that would be useful to researchers and policymakers. Currently, no data-tracking mechanism is in place at the CMM beyond Excel sheets and the documentation provided in annual reports. The existing mechanism requires a tremendous amount of work to locate critical information that is not always consistently recorded. Finally, more formal evidence to support midwifery practice such as a study related to maternal/fetal outcomes should be done. This would add to the existing body of literature related to birth outcomes across Canada [11, 12, 14, 83–85].

## Conclusion

This is the first case study in Manitoba to critically analyze barriers and facilitators that have impacted the implementation and utilization of midwifery services across the province. By identifying factors such as barriers related to structures of power and lack of a productive education program, our study has contributed new insights and knowledge about critical next steps to consider in efforts to sustain the profession in Manitoba. Our study also demonstrated how the discursive culture of midwifery politics interacts with the health care system and how policy was incongruent with the outcomes. Beyond the analysis of barriers, our study has also highlighted what has gone well and validated the profession’s progress and work related to health policy.

## Appendix A: Interview guide

### Introduction

Thank you for agreeing to participate in this interview today. I hope to gain an understanding of the historical and current factors influencing implementation and utilization of midwifery in Manitoba. Your involvement and knowledge of the midwifery scene in Manitoba will contribute greatly to this process of deconstructing these issues. Ultimately, I hope the findings will provide new information to support and help with decision-making on how to increase the utilization of midwifery services in the Manitoba.

Prior to starting this interview I would like you to understand that I have been personally and professionally involved in the midwifery scene in Manitoba for the last 6 years. It is important for me to be as objective as possible. Please answer these questions to the best of your knowledge. Please do not withhold information based on the assumption of my personal involvement with midwifery in Manitoba.

### Biographical/historical/personal background

I’d like to start with some questions about your professional background and involvement with midwifery in Manitoba.

#### Professional background/training

- What is your professional background or training?

#### Historical relationship to midwifery in Manitoba

b. When was your first involvement with implementation of midwifery in Manitoba? Tell me how you have been involved over the years to current day?
c. Why were you involved? In what capacity?

#### Current involvement with Midwifery in Manitoba

d. In what way are you currently involved with midwifery in Manitoba?
e. Are you currently involved with midwifery in other parts of Canada or the world? If yes, in what capacity?

### Factors influencing implementation and utilization of midwifery in Manitoba

I’d now like to focus on the factors influencing the implementation and utilization of midwifery in Manitoba.

1. What do you think about midwifery in Manitoba in general?
2. What do you think are the most important factors influencing implementation of midwifery in Manitoba and why?

***PROBE***: what other factors have had an impact on midwifery services in Manitoba?

### Health policy related factors to utilization of midwifery services

2. How does policy influence midwifery services? What has happened in the past with policy?
3. How does health care policy regarding a mother’s choice of health care reflect a woman’s position in society?
4. Midwifery is predominately a women’s profession, providing care to women. How do you think this has influenced its implementation?

### PROBES

#### Financial resources

Can you tell me about any financial barriers to implementation of midwifery?

How about available financial resources and their impact?

#### Educational resources

What about educational resources? How do you think the workload and organization of the profession plays a role in how midwifery services are made available?

### PROBES

#### Manpower & organization of midwives in Manitoba

What are barriers or facilitators?

### Characteristics of the Manitoba midwifery population

5. What do you think determines if a woman gets midwifery care?
6. How do you think women perceive midwifery care?
7. How do you think cultural beliefs of a woman influence her decision to seek midwifery care?

### PROBES

#### Availability of regulated midwifery services in Manitoba (Enabling factors related to utilization)

Tell me more about the impact of cultural beliefs?

#### Perceived Need of regulated midwifery services

Tell me how you think a society’s perceived need of these types of services impact the implementation and utilization?

### Consumer influence

8. How do you think consumers have influenced (or in what ways?) midwifery services in Manitoba?
9. How do you think consumer influence impacts policy related to midwifery services?
10. How do you think women have perceived midwifery services in Manitoba?
11. Why do you think women want midwifery care?

### PROBES

#### Convenience, costs, coordination, courtesy, information & quality

Can you tell me more about the importance of consumer influence?

### Future directions

12. If you could change anything about midwifery in Manitoba, what would you change?
13. What direction do you think midwifery needs to take in Manitoba?
14. What strategies are needed to improve utilization?

## Abbreviations

ACNM: American College of Nurse Midwives
BMP: Bachelor of Midwifery Program
CAM: Canadian Association of Midwives
CANSIM: Canadian Socio-Economic Information Management System
CIHI: Canadian Institute for Health information
CIHR: Canadian Institutes of Health Research
CMM: College of Midwives of Manitoba
CMRC: Canadian Midwifery Regulators Consortium
COPSE: Council on Post-Secondary Education
ERT: Winnipeg External Review Team
ICM: International Confederation of Midwives
KD & CMM: Kagike Danikobidan and College of Midwives of Manitoba
KOBP: Kanácí Otinawáwasowin Baccalaureate Program
MANA: Midwives Alliance of North America
MCHP: Manitoba Centre for Health Policy
MKO: Manitoba Keewatinowi Okimakanah
NACM: National Aboriginal Council of Midwives
PWCHE: Prairie Women’s Health Centre of Excellence
RHA: Regional Health Authority
SOGC: Society of Obstetricians and Gynaecologists of Canada
UCN: University College of the North
WHO: World Health Organization
WRHA: Winnipeg Regional Health Authority
